# Adult-Onset Polymorphic Maculopapular Cutaneous Mastocytosis

**DOI:** 10.7759/cureus.115993

**Published:** 2026-09-09

**Authors:** Shane B Devine, Veronica Alix, Jun Mendoza, Karla Adams

**Affiliations:** 1 Internal Medicine, Wright-Patterson Medical Center, Dayton, USA; 2 Allergy and Immunology, 633rd Medical Group, Langley Air Force Base, Hampton, USA; 3 Allergy and Immunology, Landstuhl Regional Medical Center, Landstuhl, DEU; 4 Allergy and Immunology, Wilford Hall Medical Center, San Antonio, USA

**Keywords:** adult-onset cutaneous mastocytosis, cutaneous mastocytosis, darier’s sign, mast cell disease, polymorphic maculopapular cutaneous mastocytosis

## Abstract

Cutaneous mastocytosis commonly manifests with maculopapular lesions. In children, the lesions tend to be large and polymorphic, while in adults, they are typically small and monomorphic. Adults who develop cutaneous manifestations are usually found to have systemic mastocytosis (SM).

In this report, we describe the case of a 42-year-old female patient who developed polymorphic hyperpigmented cutaneous lesions scattered over her chest and abdomen. Punch biopsies of two lesions were obtained and demonstrated increased perivascular and interstitial mast cells. Over the following month, she continued to develop 1-5 mm hyperpigmented macular lesions scattered over her arms, associated with intermittent flushing episodes of the face and chest, pruritus, and a positive Darier’s sign. She underwent a bone marrow biopsy, which was negative for dense infiltrates of mast cells; immature, atypical, or spindle-shaped mast cells; expression of CD2, CD25, and CD30; and the *KIT* D816V mutation. Peripheral blood was also negative for the *KIT* D816V mutation. Her basal tryptase level ranged from 2.9 ng/mL to 4.0 ng/mL, which was within normal limits. Thus, symptomatic treatment with cetirizine and montelukast was initiated.

The overall clinicopathologic correlation, including the heterogeneity of lesions, was most consistent with polymorphic maculopapular cutaneous mastocytosis (MPCM). In contrast to the typical adult presentation of monomorphic lesions and associated systemic involvement, this patient presented with polymorphic lesions and did not meet any major or minor criteria for SM. Regular allergy follow-up was indicated to monitor for potential development of systemic involvement over time.

In adult patients presenting with a polymorphic cutaneous macular eruption, it is important to keep MPCM in the differential diagnosis.

## Introduction

Mastocytosis describes a group of rare disorders characterized by the accumulation and dysregulation of mast cells [1,2]. Overall, the prevalence of mastocytosis is approximately one in 10,000 people [3]. Isolated cutaneous mastocytosis, a form in which mast cell infiltration is confined to the skin, is frequently seen in children but rarely occurs in the adult population [1,2,4,5]. Maculopapular lesions are the most common morphologic manifestation of cutaneous mastocytosis [5]. Patients may experience dermatologic symptoms, including pruritus and flushing. Darier’s sign, the development of a localized wheal upon rubbing the lesion infiltrated with mast cells, may also be present [6]. In children, the lesions tend to be large, polymorphic, and distributed over the trunk, extremities, and head [2]. In adults, the lesions are typically small, monomorphic, and distributed over the trunk and thighs [2]. Approximately 95% of adults with cutaneous manifestations are ultimately diagnosed with underlying systemic mastocytosis (SM) [2]. The criteria for diagnosing SM according to the World Health Organization and International Consensus Classification include fulfilling either one major and one minor criterion or three minor criteria [5,7]. The major criterion is defined by the presence of multifocal dense infiltrates of mast cells (>15 mast cells in aggregates) detected in sections of bone marrow or other extracutaneous organs [5,7]. The minor criteria include the presence of morphologically abnormal mast cells comprising over 25% of infiltrates in bone marrow or other extracutaneous biopsies; the aberrant expression of CD25, CD2, and/or CD30; the presence of a *KIT *D816Vmutation or other activating *KIT *mutations detected in extracutaneous tissue or peripheral blood; and an elevated baseline serum tryptase level greater than 20 ng/mL, unless there is an associated myeloid neoplasm, in which case this parameter is not valid [5,7]. 

Adult-onset polymorphic maculopapular cutaneous mastocytosis (MPCM) without systemic involvement is an uncommon presentation with limited descriptions in the literature. We present a case of a new-onset polymorphic cutaneous eruption in a 42-year-old female patient that was ultimately diagnosed as polymorphic cutaneous mastocytosis without systemic involvement. As adult cutaneous mastocytosis is usually associated with monomorphic lesions and the development of systemic disease, this case highlights an atypical presentation that may pose a diagnostic challenge. Distinguishing cutaneous disease from systemic involvement is clinically important, as SM requires additional diagnostic screening and a different therapeutic approach [5].

## Case presentation

A 42-year-old female patient with no significant medical history initially presented with increasing hyperpigmentation over her breast. Topical triamcinolone acetate 0.1% ointment twice a day for two weeks was trialed without improvement. She then developed hyperpigmented cutaneous lesions scattered over her chest and abdomen (Figures 1-2). 

**Figure 1:**
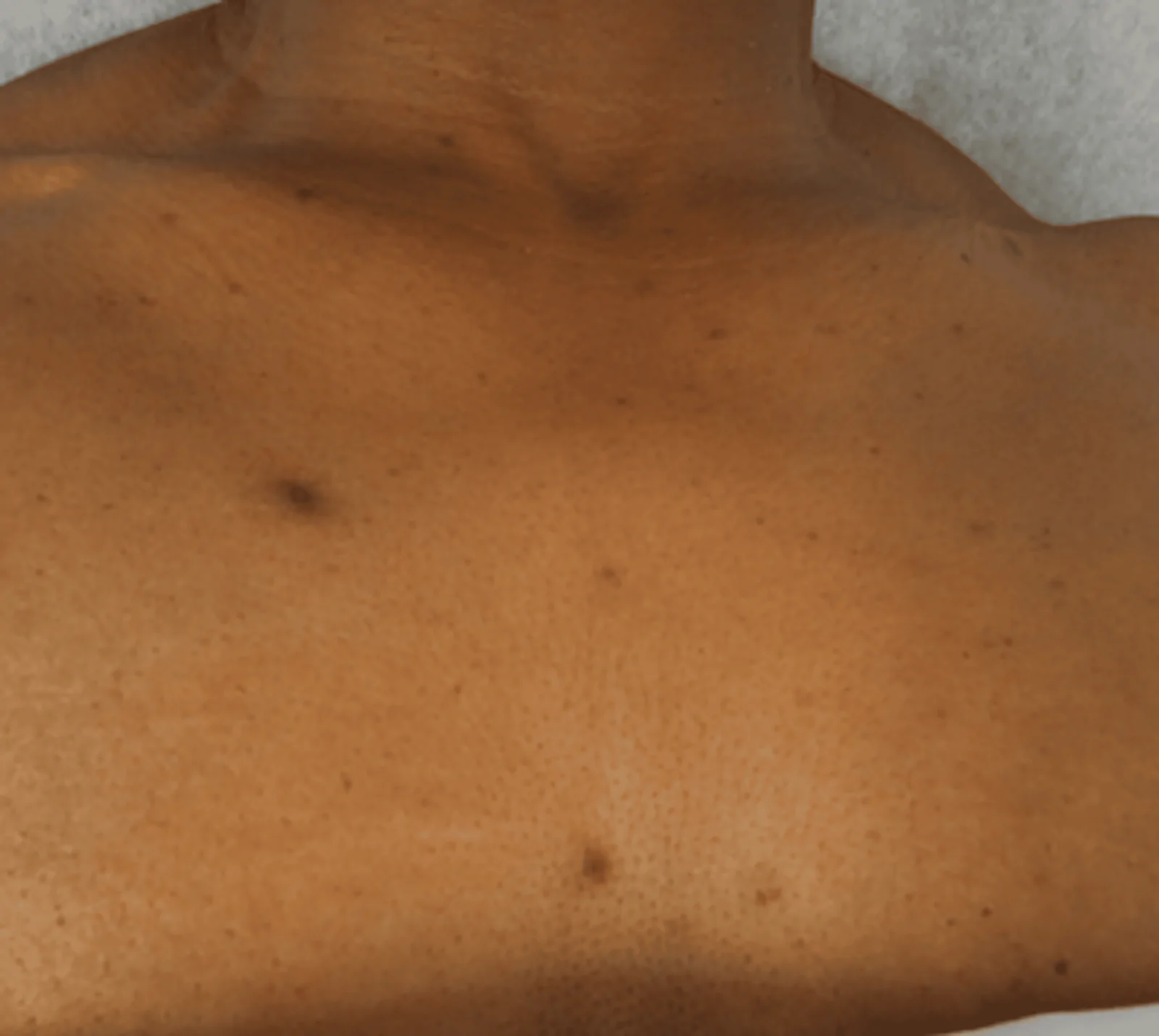
Cutaneous chest lesions Clinical image of 1-3 mm hyperpigmented cutaneous lesions scattered over the patient’s chest.

**Figure 2:**
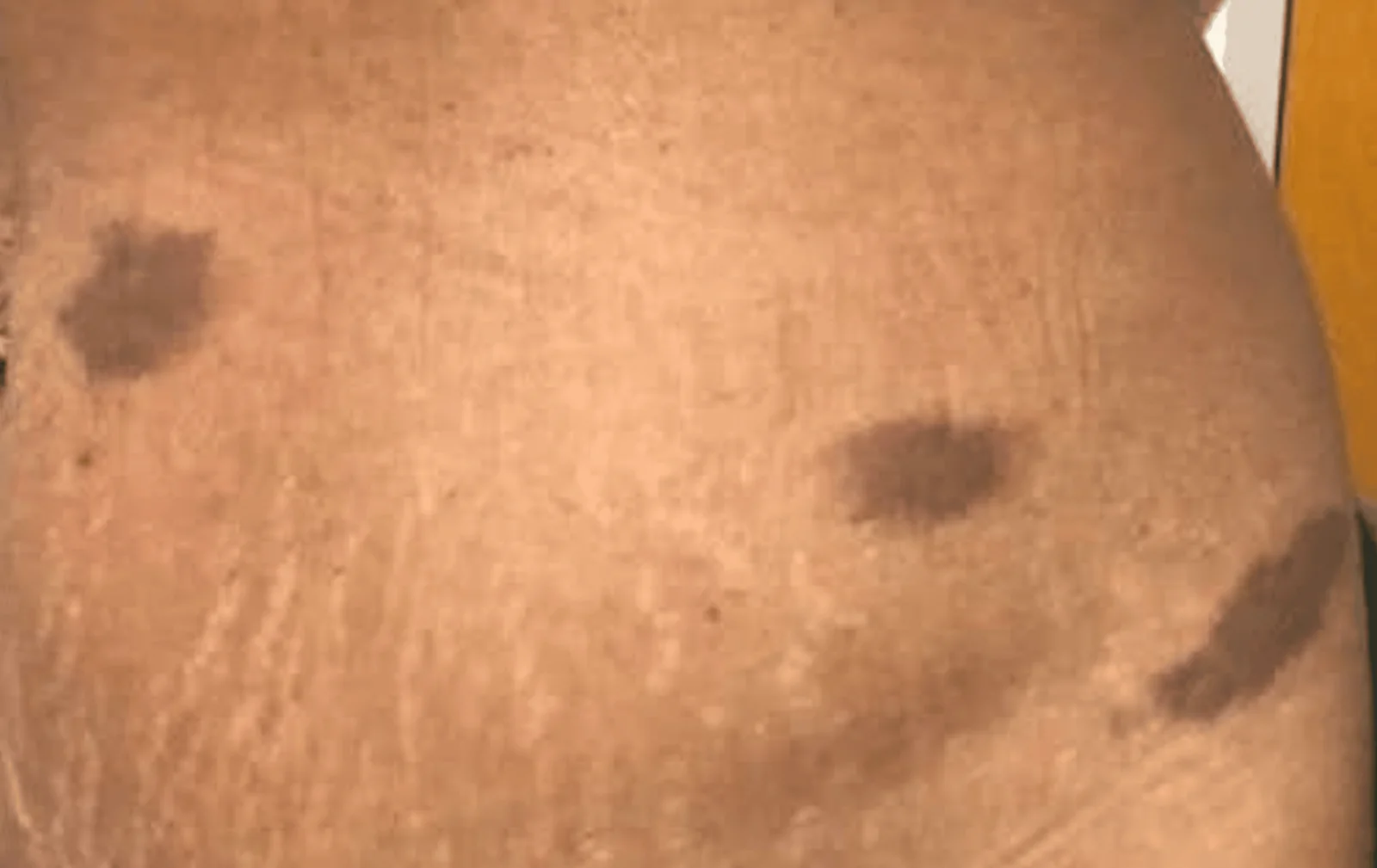
Cutaneous abdominal lesions Clinical image demonstrating 20-30 mm hyperpigmented cutaneous lesions scattered over the patient’s abdomen.

Punch biopsies of two lesions were obtained and demonstrated increased perivascular and interstitial mast cells, consistent with MPCM (Figures 3-4).

**Figure 3:**
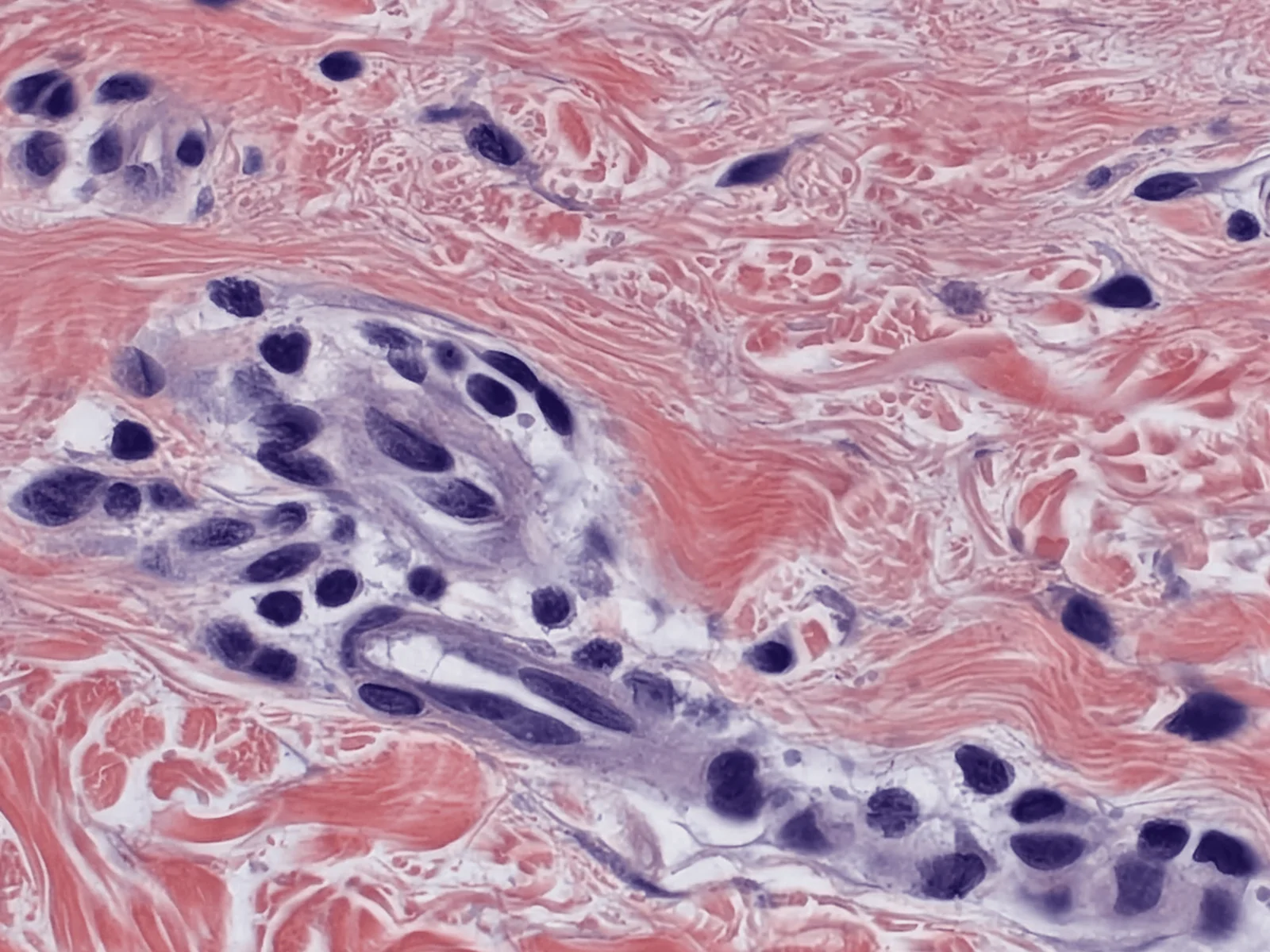
Histopathologic image of punch biopsy Hematoxylin and eosin (H&E) stain demonstrating superficial perivascular and interstitial mast cells (original magnification ×400).

**Figure 4:**
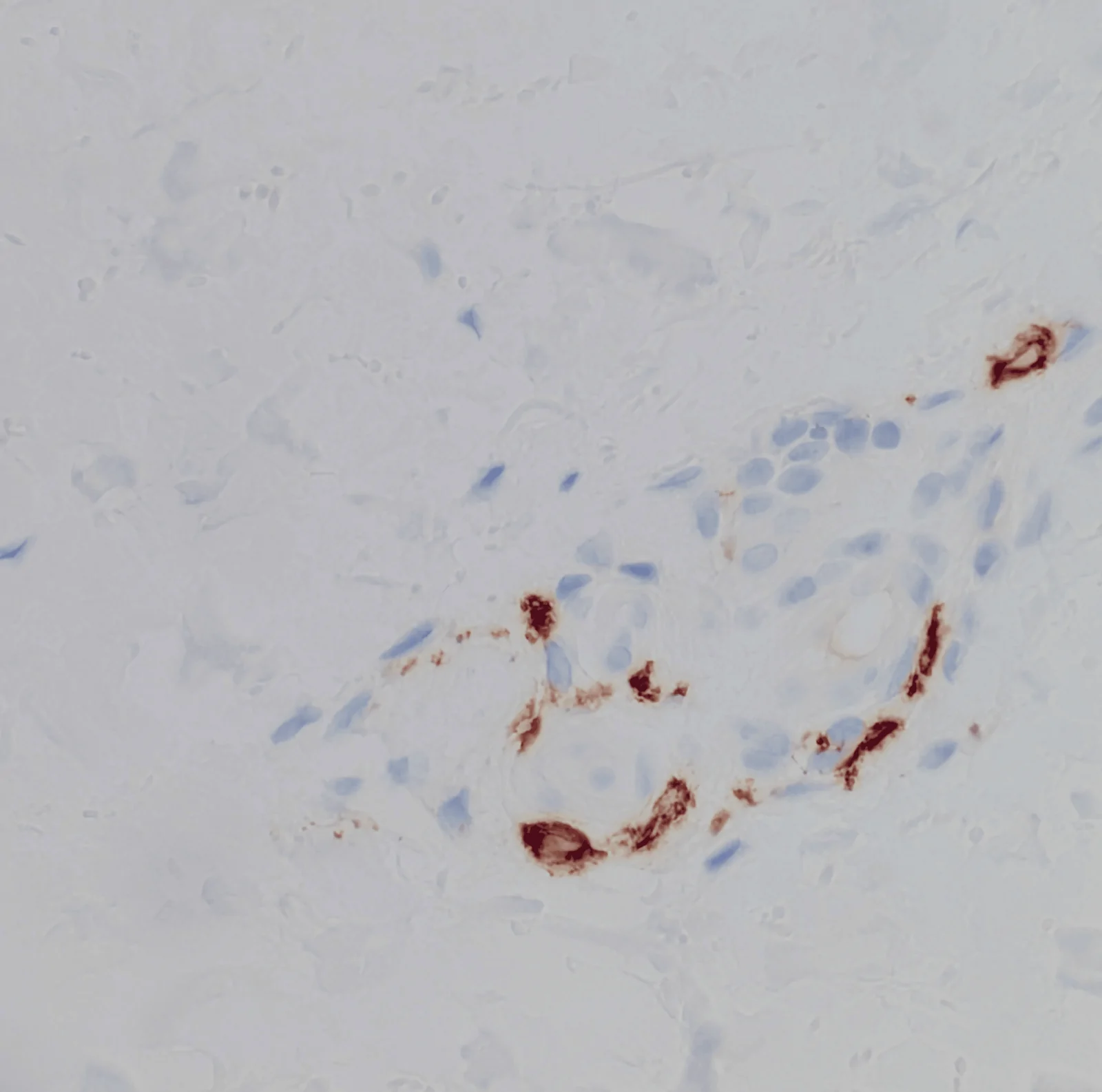
CD117 immunohistochemical staining of punch biopsy CD117 immunohistochemical stain highlighting the presence of mast cells in the perivascular and interstitial distribution (original magnification ×400). CD117 represents the c-KIT protein encoded by the *KIT *gene.

Over the following month, she continued to develop 1-5 mm hyperpigmented macular lesions scattered over her arms and face (Figure 5). During this time, she also endorsed intermittent flushing episodes involving the face and chest, accompanied by occasional pruritus and a positive Darier’s sign. These episodes were triggered by hot showers and emotional stress and were not associated with systemic symptoms, such as respiratory distress, tachycardia, palpitations, gastrointestinal distress, syncope, or anaphylaxis. Review of symptoms was otherwise negative for myalgias, decreased concentration, brain fog, fatigue, weight loss, fevers, night sweats, nausea, vomiting, diarrhea, pathologic fractures, osteopenia, or osteoporosis.

**Figure 5:**
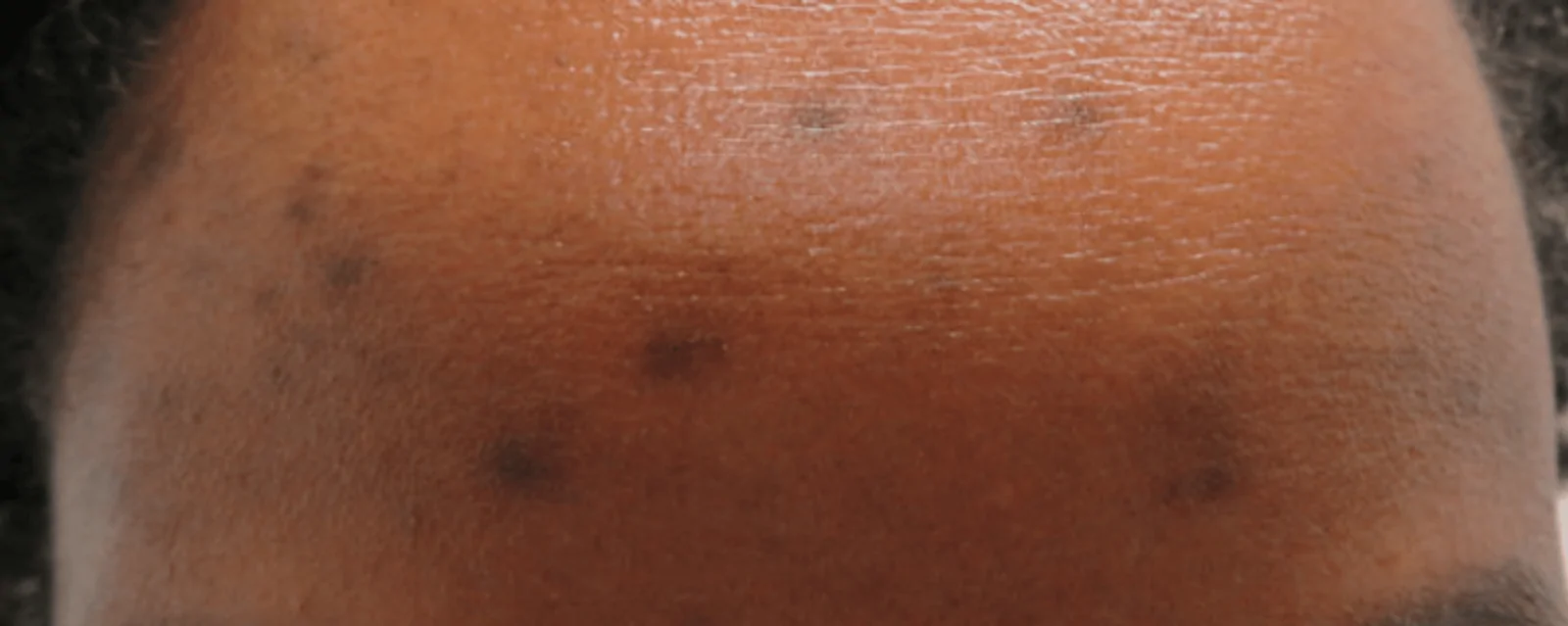
Cutaneous facial lesions Clinical image demonstrating 1-5 mm hyperpigmented macular lesions scattered over the patient’s face.

The following month, she underwent a bone marrow biopsy, which was negative for dense infiltrates of mast cells; immature, atypical, or spindle-shaped mast cells; expression of CD2, CD25, and CD30; and the *KIT *D816V mutation (Table 1). Peripheral blood testing for the *KIT* D816V mutation by droplet digital PCR (ddPCR) through ARUP Laboratories was also negative (Table 1). Her basal tryptase level ranged from 2.9 ng/mL to 4 ng/mL, which was within normal limits (Table 1).

**Table 1:** Laboratory evaluation for systemic mastocytosis Laboratory evaluation included a baseline serum tryptase level within the reference range and no detectable *KIT* D816V mutations in either the bone marrow aspirate or peripheral blood.

Lab test	Value	Reference range
Serum tryptase	4.0 ng/mL	2.2-13.2 ng/mL
*KIT* D816V mutation (peripheral blood)	Not detected	Not detected
*KIT* D816V mutation (bone marrow aspirate)	Not detected	Not detected

Symptomatic treatment with cetirizine and montelukast was thus initiated. 

## Discussion

This patient presented with polymorphic lesions on the trunk, arms, and face, associated with intermittent flushing, pruritus, and a positive Darier’s sign. She lacked other symptoms of extracutaneous mast cell activation [5]. Punch biopsy demonstrated increased perivascular and interstitial mast cells, consistent with MPCM [8]. Further evaluation for the presence of SM was negative for all major and minor criteria [5,7]. This case is notable because the heterogeneity of the patient’s lesions was most consistent with polymorphic MPCM, a subtype predominantly diagnosed in pediatric patients, making adult-onset very rare. Furthermore, the absence of systemic involvement in this patient is particularly unusual, as approximately 95% of adults with cutaneous mastocytosis have underlying SM [2]. 

Evaluation for SM includes bone marrow biopsy, testing for the *KIT* D816V mutation in both bone marrow aspirate and peripheral blood, and obtaining basal serum tryptase levels [9]. Highly sensitive PCR-based methods, such as ddPCR or allele-specific PCR, are recommended for peripheral blood testing because other sequencing methods may not be sensitive enough to detect *KIT* D816V at low allele burdens [5]. This assessment is essential to guide appropriate counseling, surveillance, and management of potential underlying SM. Patients with systemic involvement are at increased risk for life-threatening anaphylaxis and should receive counseling on trigger avoidance and the use of epinephrine autoinjectors. Additionally, these patients should undergo routine bone density screening due to the increased risk of osteoporosis [5]. Symptom-targeted therapy is recommended for all patients with mast cell diseases, but avapritinib and other targeted pharmacologic therapies may be considered for patients with SM [5]. 

Patients without systemic involvement can be treated symptomatically. Treatment is targeted at managing the mast-cell-mediated symptoms with antihistamines, histamine receptor antagonists, leukotriene receptor antagonists, or mast cell stabilizers [7]. Triggers for the release of cutaneous mast cell mediators, including emotional stress, heat, pressure, rubbing, exercise, and changes in temperature, should be avoided [7]. 

Our patient’s dermatologic symptoms of flushing and pruritus were effectively controlled with antihistamines and trigger avoidance. Given her atypical presentation, continued allergy follow-up and serial baseline serum tryptase monitoring are warranted to assess for the possible development of systemic involvement over time.

## Conclusions

In adult patients presenting with a polymorphic macular cutaneous eruption, it is important to keep MPCM in the differential diagnosis. If punch biopsy of the lesions is consistent with cutaneous mastocytosis, the evaluation for underlying SM should be completed, even in the absence of overt extracutaneous symptoms. The presence of this rare polymorphic variant in the absence of systemic symptoms underscores the unusual nature of our patient’s presentation and its diagnostic significance. Patients without present systemic involvement may be treated symptomatically with antihistamines and should undergo regular allergy follow-up with serial baseline serum tryptase monitoring to evaluate for possible systemic progression over time. 
